# Mdm2 as a chromatin modifier

**DOI:** 10.1093/jmcb/mjw046

**Published:** 2016-12-07

**Authors:** Magdalena Wienken, Ute M. Moll, Matthias Dobbelstein

**Affiliations:** 1 Institute of Molecular Oncology, Göttingen Center for Molecular Biosciences (GZMB), University Medical Center Göttingen, Göttingen 37077, Germany; 2 Department of Pathology, School of Medicine, Stony Brook University, Stony Brook, NY 11794, USA

**Keywords:** Mdm2, polycomb repressor complex, histone methylation, histone ubiquitination, EZH2

## Abstract

Mdm2 is the key negative regulator of the tumour suppressor p53, making it an attractive target for anti-cancer drug design. We recently identified a new role of Mdm2 in gene repression through its direct interaction with several proteins of the polycomb group (PcG) family. PcG proteins form polycomb repressive complexes PRC1 and PRC2. PRC2 (via EZH2) mediates histone 3 lysine 27 (H3K27) trimethylation, and PRC1 (via RING1B) mediates histone 2A lysine 119 (H2AK119) monoubiquitination. Both PRCs mostly support a compact and transcriptionally silent chromatin structure. We found that Mdm2 regulates a gene expression profile similar to that of PRC2 independent of p53. Moreover, Mdm2 promotes the stemness of murine induced pluripotent stem cells and human mesenchymal stem cells, and supports the survival of tumour cells. Mdm2 is recruited to target gene promoters by the PRC2 member and histone methyltransferase EZH2, and enhances PRC-dependent repressive chromatin modifications, specifically H3K27me3 and H2AK119ub1. Mdm2 also cooperates in gene repression with the PRC1 protein RING1B, a H2AK119 ubiquitin ligase. Here we discuss the possible implications of these p53-independent functions of Mdm2 in chromatin dynamics and in the stem cell phenotype. We propose that the p53-independent functions of Mdm2 should be taken into account for cancer drug design. So far, the majority of clinically tested Mdm2 inhibitors target its binding to p53 but do not affect the new functions of Mdm2 described here. However, when targeting the E3 ligase activity of Mdm2, a broader spectrum of its oncogenic activities might become druggable.

## Mdm2 and its distinct roles in p53 regulation

The E3 ubiquitin ligase Mdm2 is the central physiological antagonist of the tumour suppressor p53 (Li and Lozano, 2013). Mdm2 affects p53 stability by targeting it for proteasomal degradation (Haupt et al., 1997). Furthermore, Mdm2 inhibits p53-mediated transactivation and also controls its cellular localization (Momand et al., 1992; Roth et al., 1998; Li et al., 2003). Nearly all human tumours have lost normal p53 functions. In the absence of p53 mutations, such a loss can be brought about, for example, by supraphysiological Mdm2 level and activity (Muller and Vousden, 2013). Several drugs that abrogate the Mdm2−p53 interaction have entered clinical trials (Wade et al., 2013).

Nearly 20 years ago, Lozano and Bradley groups developed mice with targeted disruptions of both p53 and Mdm2 (Jones et al., 1995; Montes de Oca Luna et al., 1995). Conversely, loss of Mdm2 alone resulted in early embryonic lethality due to massive induction of p53 and embryonic death via apoptosis. Combining the deletions of p53 and Mdm2 reversed this phenotype completely, enabling live mice. At first glance, and like p53^−/−^ mice, the p53^−/−^;Mdm2^−/−^ mice were not grossly affected in their development, although both genotypes were associated with cancer. It was concluded that the main function of Mdm2 consists in the regulation of p53 (Jones et al., 1995; Montes de Oca Luna et al., 1995). However, several investigations shed doubt on this black-and-white picture of p53 and Mdm2.

First, subsequent reports stated irregular breeding patterns and developmental defects of both p53^−/−^ and p53^−/−^;Mdm2^−/−^ animals (Armstrong et al., 1995; Montes de Oca Luna et al., 1995; Sah et al., 1995). For instance, Armstrong et al. (1995) as well as Sah et al. (1995) reported that p53 knockout mice displayed increased embryonic lethality due to insufficient closure of the neural tube and exencephaly, despite the overall assumption that loss of p53 did not alter development and survival of the animals. Furthermore, in one of the first papers describing the p53^−/−^;Mdm2^−/−^ mice, very small litter numbers and few pups within a littler were mentioned. The mechanistic reasons are unknown (Montes de Oca Luna et al., 1995). Second, multiple human tumours were identified, which carry high levels of Mdm2 even in the absence of wt p53 or express Mdm2 isoforms that are no longer able to bind to and regulate p53 (Cordon-Cardo et al., 1994; Sigalas et al., 1996). Third, mammary gland-specific overexpression of Mdm2 in mice abrogated normal mammary gland development in a p53-independent manner and led to the formation of mammary tumours (Lundgren et al., 1997). These findings argued that Mdm2 is carrying out additional functions beyond the mere regulation of p53. This might be due to the ability of Mdm2 to regulate gene expression, even in the absence of p53, as described below.

## Mdm2 acts on gene expression and chromatin modifications in a p53-dependent or p53-independent way

Mdm2 has long been known to influence p53-mediated gene expression and even alter target gene selectivity. Studies, also including those in the Mdm2 homologue Mdmx, identified differences in the regulation of p53-responsive genes. Whereas *Bax* gene expression was reduced by Mdm2, Mdmx preferentially diminished the expression of the *CDKN1A/p21* gene by p53 (Chavez-Reyes et al., 2003). *In vivo*, the differential regulatory impact on p53 led to an embryonic lethality in Mdm2^−/−^ mice at 6.5 dpc due to apoptosis, whereas Mdmx^−/−^ mice died at 7.5 dpc due to cell cycle arrest. Furthermore, *in vitro* studies with fibroblasts generated from p53^−/−^;Mdm2^−/−^ as well as p53^−/−^;Mdmx^−/−^ mice identified overlapping but non-coinciding sets of p53-responsive genes (Parant et al., 2001; Barboza et al., 2008). One way of how Mdm2 represses p53-responsive genes consists in its direct interaction with p53 on its target gene promoters, leading to an inhibition of transcription machinery assembly (Arva et al., 2005). Furthermore, the recruitment and interaction with the histone methylases SUV39H1 and EHMT1 is thought to facilitate the generation and maintenance of repressive chromatin (Chen et al., 2010).

Already in 1997, the Tjian laboratory showed that Mdm2, when associating with naked DNA, represses gene expression also in a p53-independent way. In their study, a fusion protein of Mdm2 and a specific DNA-binding domain interfered with the basal transcription machinery through direct interaction with the TFIIE small subunit and the TATA box binding protein (Thut et al., 1997). Subsequent reports hypothesized that Mdm2 directly influences gene expression through interaction with and recruitment of specific transcription factors (e.g. E2F1, NFΚB, and TGFβ signalling) and DNA damage response proteins, as well as by modification of histones and remodelling of nucleosome structures (Martin et al., 1995; Bennett-Lovsey et al., 2002; Biderman et al., 2012).

### Mdm2 affects genome integrity

Mdm2 is capable of interacting with the MRN complex, composed of Mre11, Rad50, and Nbs1, which functions in DNA double-strand break repair. In studies from the Eischen laboratory, Mdm2 co-localizes with Nbs1 to sites of DNA damage and delays repair mechanisms (Alt et al., 2005). Interestingly, this function is independent of p53 and does not require the Mdm2 RING domain function. This activity was also observed for the Mdm2-homologue Mdmx (Carrillo et al., 2015). In addition, it is very possible that Mdm2 can modulate the DNA damage response and DNA repair by modifying chromatin, as described below.

### Mdm2 regulates chromatin dynamics and gene expression

Early analyses demonstrated that Mdm2 associates with chromatin on endogenous promoter sequences of p53 target genes. For these experiments, targeted chromatin immunoprecipitation (ChIP) was performed in cells with high endogenous Mdm2 levels (e.g. SJSA, Minsky and Oren, 2004), after forced Mdm2 overexpression (Tang et al., 2008), and in cells expressing normal Mdm2 levels (White et al., 2006). Unfortunately, there is no global Mdm2 ChIP-sequencing data set available to date. In our experience, the ChIP yield is too small to allow deep sequencing, perhaps due to the low abundance of endogenous Mdm2 in most cells. However, it is hoped that with refinement of cross-linking strategies, antibody strength, and sequencing technology, we might soon get a glimpse on the genome-wide distribution of Mdm2 on chromatin.

Since Mdm2 recruitment to p53 target genes was not detected in p53-null cells in previous studies, its association with chromatin was thought to completely depend on p53. Furthermore, acetylation of p53 abolished the binding of Mdm2 to these promoters (Tang et al., 2008). The authors of this study proposed that Mdm2 represses gene expression through the recruitment of histone deacetylases, especially HDAC1 (Ito et al., 2002), and the inhibition of histone acetyl transferases (HATs) (Ito et al., 2001; Legube et al., 2002). Transactivation of gene expression by p53 requires acetylated p53 as post-transcriptional modification pattern (Ito et al., 2001). p53 acetylation is carried out by the HAT Tip60 or p300/CBP, and both of them have been shown to be inhibited or degraded via Mdm2 (Ito et al., 2001; Legube et al., 2002). Furthermore, interaction of Mdm2 with HDAC1 mediates p53 deacetylation and inhibition of transcription (Ito et al., 2002). The interactions with HATs and HDAC1 were reasoned enough to speculate that Mdm2 might be directly involved in the acetylation/deacetylation of histone proteins. Acetylation of histones at lysine residues is associated with a decrease of positive charge, opening nucleosome packaging, and facilitating gene expression (Grunstein, 1997). The recruitment of HDACs by Mdm2 and the inhibition of HATs could—in addition to the regulation of p53—support transcriptionally silent chromatin, although this remains to be confirmed experimentally.

Mdm2 was also found to support repressive chromatin structures by enhancing trimethylation of Histone 3 at lysine 9 (Chen et al., 2010; Cross et al., 2011). Mdm2 interacted with the histone methyl transferases SUV39H1 and EHMT1 and facilitated their interaction with p53 on p53 target genes. In this way, target gene expression was repressed by histone methylation. Moreover, p53 was inhibited through methylation of its residue lysine 373 (Chuikov et al., 2004). However, more recent publications challenged this result. There is evidence that Mdm2 rather targets SUV39H1 for lysine 87 polyubiquitination and subsequent proteasomal degradation (Bosch-Presegué et al., 2011). This then relieves p53 target gene repression in a stress-induced, p53-activated cellular context. Indeed, upon p53 activation, SUV39H1 was negatively regulated at the post-translational level by Mdm2 and at the transcriptional level by the p53 target gene product p21 (Mungamuri et al., 2012, 2016).

These at least partially contradictory findings could be due to the use of different experimental systems. Whereas Mungamuris et al. (2016) mainly focused on cancer cell lines, Chen et al. (2010) also used mouse embryonic fibroblasts (MEFs) derived from p53^−/−^;Mdm2^−/−^ mice. So far, our knowledge on Mdm2 functions in non-transformed cells is limited. Thus, it is possible that histone methylation and gene repression by Mdm2 predominate in one context, whereas the decay of histone methylases is more pronounced in the other. The authors detected distinct Mdm2-dependent gene regulatory patterns when comparing primary cells, stem cells, and cancer cells in high-throughput transcriptome studies, suggesting system-dependent differences in Mdm2 functions (Wienken et al., 2016). Furthermore, while Wienken et al. (2016) focused mainly on the gene repressive function of Mdm2 and its role in development, Riscal et al. (2016) shortly thereafter described a p53-independent role of Mdm2 in gene activation to modulate metabolic activity, again highlighting the importance of context and specificity for Mdm2 function.

Besides histone acetylation and methylation, Mdm2 can also modulate histone ubiquitination. In particular, Minsky and Oren (2004) reported that Mdm2 was not only able to directly interact with certain histones, but also ubiquitinate histone H2A (*in vivo*) and H2B (*in vitro*). This function was dependent on the Mdm2 RING domain and was also associated with its gene repressive function, e.g. on the p53 target gene *CDKN1A/p21*. More recent data, however, strongly suggest that at least the H2B-ub mark is more often associated with actively transcribed genes, facilitating elongation by allowing the progress of RNA Pol II through the gene body. The ubiquitination mark is established through the ubiquitin ligases RNF20/40 and was identified to support stem cell differentiation. Its loss is often associated with malignancy (Kim et al., 2005; Karpiuk et al., 2012). Therefore, it would be at least somewhat counter-intuitive if Mdm2 supported H2A ubiquitination *in vivo*. On the other hand, H2A ubiquitination, specifically H2AK119ub1, is most frequently associated with gene repression. Histone 2A lysine 119 (H2AK119) monoubiquitination can be mediated by the polycomb group (PcG) family member RING1B/RNF2 and then maintains gene repression and supports an undifferentiated stem cell phenotype, sometimes contributing to malignancy (Sparmann and van Lohuizen, 2006). In a recently published report by our laboratory, we identified a crucial role for Mdm2 in stemness maintenance and cancer cell survival, via stabilization of H2AK119ub1 and the closely associated trimethylation of histone 3 lysine 27 (H3K27) (Wienken et al., 2016), as detailed below.

## iPSC generation is supported by Mdm2 even in the absence of p53

In 2009, a number of studies demonstrated that the generation of induced pluripotent stem cells (iPSCs) via the Yamanaka protocol was inhibited by p53 or by its downstream effector CDKN1A/p21 (Hong et al., 2009; Kawamura et al., 2009). These findings were quite surprising and urged our laboratory to ask whether the iPSC system might also reveal an unknown role of Mdm2 in stemness and differentiation. Indeed, we found that the presence of Mdm2 strongly enhances the reprogramming efficacy in p53^−/−^ knockout MEFs (Wienken et al., 2016). In parallel, a specific gene expression pattern was found to depend on the presence of Mdm2, specifically its RING finger domain. As indicated by gene set enrichment analysis, many identified Mdm2 target genes are known key regulators of pluripotency. H3K27me3 is often observed at these genes as well, strongly suggesting their regulation through the PcG family.

### The PcG family shares gene regulatory functions with Mdm2

The PcG family was originally defined in *Drosophila.* These gene products were grouped together because loss of any of these proteins resulted in a specific homeotic transformation—additional sex combs on male *Drosophila* legs (Jürgens, 1985). Homeotic transformation was caused by upregulation of homeotic (Hox) transcription factors that regulate early fly development (Lewis, 1978).

The PcG family can be further subdivided into several polycomb repressive complexes (PRCs), among which PRC1 and PRC2 are well characterized. Through their enzymatic components EZH2 (PRC2) and RING1B (PRC1), these complexes mediate H3K27 trimethylation and H2AK119 monoubiquitination, respectively. These post-translational histone modifications support a compacted and transcriptionally silent chromatin structure that represses differentiation genes in stem cells and during early development (Simon and Kingston, 2013; Breiling, 2015). Upon differentiation, PRC levels decrease and the expression of early differentiation genes is induced (Boyer et al., 2006). Residual PRC proteins repress stemness maintenance genes in differentiated cell types and support a specific lineage gene profile through target gene regulation (Chou et al., 2011; Breiling, 2015). In concordance, Onder et al. (2012) identified a crucial role for PcG members in the generation of iPSCs. More recently, we found an analogous role for Mdm2, independent of p53 (Wienken et al., 2016).

In agreement with Hox transcription factors representing an important gene family regulated by the PcG, we detected the repression of several Hox genes by Mdm2 in p53^−/−^ MEFs. Through inhibition of EZH2 with the selective EZH2 inhibitor EPZ6438, we confirmed the direct repression of these Hox genes by PRC2 (Knutson et al., 2013). Moreover, we detected less prominent repression of overlapping gene sets in p53^−/−^Mdm2^−/−^ compared with p53^−/−^ MEFs, arguing that Mdm2 and EZH2 are acting in an epistatic fashion.

### Mdm2 and PRC2 share a gene regulatory profile that enhances stemness and proliferation

The importance of the PcG family in the maintenance of stemness was further verified by loss-of-function studies on differentiation. Apart from a few lineages, the presence of PRCs suppresses differentiation by repressing lineage-specific genes (Chou et al., 2011). Surprisingly, we identified a similar function for Mdm2 in osteoblast differentiation. Loss of Mdm2—even in the absence of p53—accelerated the differentiation of human mesenchymal stem cells (hMSCs) into osteoblasts. The same phenotype was detected upon loss of EZH2. The stem maintenance function of Mdm2 in hMSCs was associated with its capability to repress a large group of genes, half of which were also targeted by EZH2. Among the co-repressed genes were the early osteoblast differentiation markers *ALPL*, *BGLAP*, and *BMP4* as well as the late differentiation gene *IGF2* (Twine et al., 2014). In contrast, Mdm2 was recently described to support the differentiation of adipocytes from MSCs (Hallenborg et al., 2012), whereas it prevents osteoblast differentiation (Hemming et al., 2014). Thus, adipocyte differentiation and stemness are both supported by PRC2 and Mdm2.

A p53-independent and shared gene regulatory function of Mdm2 and EZH2 was not only detected in hMSCs but also in the colon cancer cell line HCT116 and the breast cancer cell line MCF7 (Wienken et al., 2016). In fact, 70% of all genes regulated by Mdm2 in HCT116 p53^−/−^ cells were also targets of EZH2. Examples of PRC2 target genes that were co-regulated by Mdm2 include *CXCR4*, *KLF2*, and *FBXO32*. Furthermore, proliferation and survival of colon, breast, bone, and pancreatic tumour cell lines were decreased upon loss of Mdm2 or EZH2, and this effect was not dependent on p53. The identification of a common gene regulatory network governed by EZH2 and Mdm2 in primary cells, stem cells, and cancer cells raised the question of an interactive relationship between the two proteins.

### Mdm2 is recruited to its target genes through direct interaction with PRC2 proteins

Co-immunoprecipitation of endogenous and overexpressed proteins as well as co-localization studies revealed direct complex formation of Mdm2 with the PRC2 proteins EZH2 and SUZ12, conserved between the murine and the human systems. The interaction domain was mapped to an N-terminal region between the p53-binding domain and acidic domain of Mdm2. In concordance, the p53-reactivating drug Nutlin-3a, which selectively interacts with the p53-binding domain of Mdm2, did not abrogate the interaction of Mdm2 and EZH2 (Müller et al., 2007). ChIP indicated that the interaction with EZH2 recruits Mdm2 to previously identified Mdm2/PRC2 target gene promoters. Interestingly, the enrichment of Mdm2 was selectively detected on Mdm2/PRC2 co-regulated genes but not on other PRC2 target genes.

### Mdm2 enhances the repressive chromatin modifications H3K27me3 and H2AK119ub1

Since we had observed an interactive gene regulatory network of Mdm2 with members of the PcG, we also expected changes to the associated histone modifications. Most interestingly, Minsky and Oren (2004) had already shown that Mdm2 is able to ubiquitinate H2A and H2B *in vitro*, possibly repressing transcription *in vivo*. By using targeted ChIP, we detected reduced H3K27me3 and H2AK119ub1 levels on Hox genes in Mdm2^−/−^ MEFs. Further genome-wide analysis via ChIP sequencing (ChIP-Seq) revealed a global loss of transcription start site-associated H2AK119ub1, and a less pronounced but overlapping loss of H3K27me3 upon removal of Mdm2. As monitored by GREAT analysis, which provides a method of computing functional annotation based on the occupancy of specific genomic regions, the overlapping regions where H2AK119ub1 and H3K27me3 were lost were associated with genes involved in stemness and development (McLean et al., 2010). Moreover, when quantifying mRNA levels upon removal of Mdm2 by RNA-Seq analysis, differentially expressed genes showed similar enrichments of GO terms as in the ChIP-Seq analysis (McLean et al., 2010).

## Depletion of Ring1B and Mdm2 is synthetic lethal

The chromatin functions of Mdm2 were dependent on a functional RING finger domain. Thus, we suspected a direct role of Mdm2 in ubiquitinating H2AK119. According to previous reports, the primary H2AK119 E3 ligase is the PRC1 protein RING1B (Ben-Saadon et al., 2006; Eskeland et al., 2010). This raised the question whether Mdm2 and RING1B pursue distinct or shared functions in the regulation of their target genes (Wang et al., 2004). Indeed, we identified several Hox genes to be repressed by both Mdm2 and RING1B. Strikingly, loss of both factors induced their expression far more than each single loss. Furthermore, the proliferation of primary cells as well as cancer cells was impaired upon simultaneous loss of Mdm2 and RING1B, suggesting synthetic lethality.

In summary, we described a new p53-independent role for Mdm2 in gene repression, which maintains stemness and supports tumour cell survival (Wienken et al., 2016). Mdm2 enhances the repressive chromatin modifications H3K27me3 and H2AK119ub1 and complements PRC1 E3 ligase activity, leading to synthetic lethality when Mdm2 and Ring1B are co-depleted.

### How does an organism develop in the absence of Mdm2?

The generation of p53^−/−^;Mdm2^−/−^ animals that developed normally initially implied that Mdm2 does not have any important p53-independent functions during embryonic development and the maintenance of an organism (Jones et al., 1995; Montes de Oca Luna et al., 1995; Chua et al., 2015). However, several studies have now discovered p53-independent cellular functions of Mdm2, which are, among others, important for the development of fat and bone tissue from MSCs (Hallenborg et al., 2012; Wienken et al., 2016). The importance of these p53-independent functions could also explain why—according to Montes de Oca Luna et al. (1995)—there are decreased numbers of litters and only few pups within a litter of p53^−/−^;Mdm2^−/−^ mice.

Still, the question remains why there are p53^−/−^;Mdm2^−/−^ animals surviving with near-normal development. One hypothesis is that particular functions of Mdm2 might only be revealed under stress conditions, as also suggested by Marine and Lozano (2010). According to this model, the activation of the p53−Mdm2 signalling axis is mainly dependent on stress impulses, such as genotoxicity, infections and inflammation, hypoxia, or temperature shifts. Laboratory animals are normally not facing extensive stress, which could disguise important Mdm2 functions due to system inactivity. *In vitro* cell systems, in contrast, are characterized by cellular stress, e.g. through hyperoxia, growth on plastic, or permanent stimulation by serum. This is further intensified by reprogramming and differentiation procedures, as well as transient transfection. To clarify this hypothesis, it would be necessary to monitor the development of p53^−/−^;Mdm2^−/−^ animals under specified stress conditions.

### What might be the evolutionary advantage of a joint Mdm2−PcG network in a cell?

Each cell is constantly facing a multitude of genotoxic stresses, including UV irradiation, toxins, and reactive oxygen species. Different signalling systems ensure that damage is recognized and repaired. The p53 system is a central player in this cascade, inducing apoptosis if the damage is too extensive, and otherwise cell cycle arrest to facilitate damage repair (Jackson and Bartek, 2009). As an early event during this stress response, Mdm2 levels and activity are decreased, leading to the activation of p53 (Vogelstein et al., 2000; Vousden, 2000). Based on our findings, we speculate that low levels of Mdm2 might support the stress response through an additional mechanism, i.e. the upregulation of genes that are normally repressed by PcG members. In this way, differentiation signalling could increase, while cell proliferation is decreased. This could provide a window of time to allow DNA repair, or alternatively at least keep stem cells from proliferating in the presence of mutations.

### How does Mdm2 fit into the PcG network?

Recent publications covering the PcG hierarchy changed the initial model accepted for the last decade and revealed the presence of at least two PRC1 subtypes with distinct functions (Comet and Helin, 2014). Canonical PRC1 (cPRC1) is characterized by the presence of chromodomain containing CBX proteins that bind existing H3K27me3 and therefore recruit cPRC1 to PRC2 target sites. This PRC1 variant is less efficient in ubiquitinating H2A at K119 and may mediate chromatin compaction through an ubiquitin-independent (but currently unknown) mechanism (Eskeland et al., 2010; Blackledge et al., 2014). On the other hand, variant PRC1 (vPRC1) is characterized by the presence of RYBP instead of CBX proteins. vPRC1 binds to target genes in a H3K27me3-independent way and readily ubiquitinates H2AK119. It is possible that vPRC1 recognizes its target genes through the interaction of its association partner KDM2B with non-methylated CpG islands. Subsequently, H2BK119 ubiquitination may help to recruit PRC2, thereby reversing the classic PcG hierarchy (Blackledge et al., 2014; Kalb et al., 2014).

**Figure 1:**
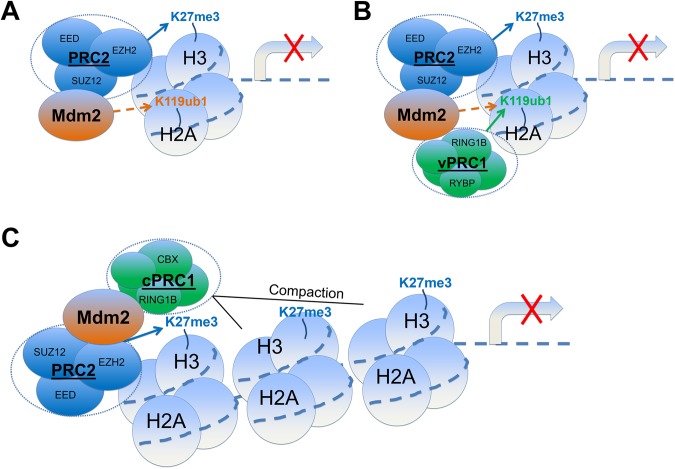
Hypothetical, non-mutually exclusive models of the Mdm2−PcG interactive network. Mdm2 is interacting with several members of the PcG protein family and enhances both H3K27 trimethylation and H2AK119 monoubiquitination. H3K27me3 may be enhanced by Mdm2 through activity changes of EZH2, or indirectly because PRC2 activity is enhanced by adjacent H2AK119ub1. H2AK119 could be directly ubiquitinated by Mdm2, but additional or alternative working models are also conceivable. (**A**) Mdm2 could provide E3 ubiquitin ligase function on distinct PRC2 target gene loci, making the maintenance of gene repression by H2AK119ub1 independent of the presence of PRC1. What argues against this model as the sole scenario is our observation that the global H2AK119ub1 levels in cells severely drop upon RING1B depletion, even when Mdm2 is still present. (**B**) The second and third working models are based on quite recent observations summarized by Comet and Helin (2014). According to their studies, PRC1 is characterized by a canonical or variant form containing CBX or RYBP, respectively. Mdm2 might facilitate the interaction between vPRC1 and PRC2 through simultaneous interaction with RING1B/RYBP and EZH2/SUZ12. Although we did not observe a decrease in RING1B on PRC2 target promoters upon removing Mdm2, it does not exclude that certain PRC1 species (like canonical and variant) might still associate with Mdm2-bound chromatin. Mdm2 would have two functions according to this model: bridging PRC1 and PRC2, as well as H2AK119 ubiquitination. (**C**) Similar to **B**, Mdm2 might bind to specific PRC1 subspecies, either canonical or variant. Here, Mdm2 could facilitate the recruitment of cPRC1 to PRC2 or H3K27me3. Instead of ubiquitinating H2AK119, this complex was reported to act by compacting chromatin through unknown mechanisms (Eskeland et al., 2010). We speculate that these hypothetical mechanisms may act together and may depend on each other, as is often the case when several repressive modifications trigger chromatin compaction.

The different PRC constellations substantially increase the complexity of chromatin modifications. This complexity is further enhanced by a multitude of PRC interaction partners, e.g. JARID2 and PCL proteins (Simon and Kingston, 2013). Mdm2 also belongs to this group, as revealed by its binding to PRC2 (Wienken et al., 2016) and by its interactions with the PcG proteins RYBP and RING1B (Fåhraeus and Olivares-Illana, 2014). Through interactions with EZH2 and SUZ12, Mdm2 could provide an E3 ubiquitin ligase function to PRC2, enabling it to act PRC1-independently (Figure 1A). According to the data from our laboratory (Wienken et al., 2016) and others (Ben-Saadon et al., 2006), H2AK119ub1 levels are clearly dependent on RING1B at the majority of genomic regions. Still, it remains possible that a subset of genes is ubiquitinated on H2A by Mdm2-associated PRC2, establishing an alternative, PRC1-independent route of H2AK119 ubiquitination.

Moreover, interaction of RYBP with Mdm2 could facilitate the cooperation between PRC2 and vPRC1, with Mdm2 forming a physical bridge between the two and coupling H3K27 trimethylation with H2AK119 monoubiquitination (Figure 1B). In our hands, the overall association of PRC1 and PRC2 with chromatin was not grossly affected by the presence of Mdm2. However, we do not know whether only specific complexes like variant and cPRC1 are affected by Mdm2 in their binding to specific chromatin regions. Likewise, in a third scenario, Mdm2 could couple PRC2 and cPRC1 through the binding of RING1B, thereby enhancing both H3K27me3 and chromatin compaction (Figure 1C).

Mdm2 might also interfere with PcG enzymatic activity by ubiquitinating PRC members. Indeed, several PRC members are known to be targeted in activity and stability by ubiquitination (Ben-Saadon et al., 2006). However, these models remain to be tested. As a negative regulator of gene expression, Mdm2 was also described to activate histone deacetylase 1 (HDAC1) and inhibit p300/CBP and other effectors of transcriptional activation (Biderman et al., 2012). These networks could further versify the PcG controlled gene profile, e.g. through coupling repressive chromatin modifications with the removal of activating acetylations.

## Possible therapeutic implementations of Mdm2 chromatin functions

The majority of therapeutic compounds targeting Mdm2 are designed to competitively bind to the deep hydrophobic p53-binding cleft of Mdm2, abrogating the interaction of the two proteins and thereby activating p53-mediated tumour suppression (Wade et al., 2013). Our data strongly argue that these inhibitors will fail to target p53-independent but yet tumour-promoting activities of Mdm2. Along this line, several compounds that target the E3 ligase activity instead of the p53-binding domain of Mdm2, e.g. Mel23/24, HLI98, and JNJ-26854165, showed p53-independent cytotoxicity and led to cell cycle arrest and apoptosis of HCT116 p53^−/−^ cells (Yang et al., 2005). Most of the clinical trials that have already been launched with Mdm2-inhibiting compounds involved drugs that target the p53-binding pocket. However, proof of clinical efficacy has not been reported with these compounds so far. Thus, compounds that inhibit the Mdm2 E3 ubiquitin ligase activity, or otherwise destabilize the Mdm2 protein, will be of utmost interest for clinically successful strategies in cancer therapy.
